# M1 muscarinic acetylcholine receptor dysfunction in moderate Alzheimer’s disease pathology

**DOI:** 10.1093/braincomms/fcaa058

**Published:** 2020-05-12

**Authors:** Jee Hyun Yi, Daniel J Whitcomb, Se Jin Park, Celia Martinez-Perez, Saviana A Barbati, Scott J Mitchell, Kwangwook Cho

**Affiliations:** f1 Bristol Medical School, Faculty of Health Sciences, University of Bristol, Bristol BS1 3NY, UK; f2 Department of Life and Nanopharmaceutical Sciences, Kyung Hee East-West Pharmaceutical Research Institute, College of Pharmacy, Kyung Hee University, Seoul 130-701, Korea; f3 UK Dementia Research Institute at King’s College London, Department of Basic and Clinical Neuroscience, Institute of Psychiatry, Psychology and Neuroscience, King’s College London, London SE5 9NU, UK

**Keywords:** Alzheimer’s disease, M1 muscarinic acetylcholine receptor (mAChR), metabotropic glutamate receptor 5 (mGluR5), recognition memory

## Abstract

Aggregation of amyloid beta and loss of cholinergic innervation in the brain are predominant components of Alzheimer’s disease pathology and likely underlie cognitive impairment. Acetylcholinesterase inhibitors are one of the few treatment options for Alzheimer’s disease, where levels of available acetylcholine are enhanced to counteract the cholinergic loss. However, these inhibitors show limited clinical efficacy. One potential explanation for this is a concomitant dysregulation of cholinergic receptors themselves as a consequence of the amyloid beta pathology. We tested this hypothesis by examining levels of M1 muscarinic acetylcholine receptors in the temporal cortex from seven Alzheimer’s disease and seven non-disease age-matched control brain tissue samples (control: 85 ± 2.63 years old, moderate Alzheimer’s disease: 84 ± 2.32 years old, *P*-value = 0.721; eight female and six male patients). The samples were categorized into two groups: ‘control’ (Consortium to Establish a Registry for Alzheimer’s Disease diagnosis of ‘No Alzheimer’s disease’, and Braak staging pathology of I–II) and ‘moderate Alzheimer’s disease’ (Consortium to Establish a Registry for Alzheimer’s Disease diagnosis of ‘possible/probable Alzheimer’s disease’, and Braak staging pathology of IV). We find that in comparison to age-matched controls, there is a loss of M1 muscarinic acetylcholine receptors in moderate Alzheimer’s disease tissue (control: 2.17 ± 0.27 arbitrary units, *n* = 7, Mod-AD: 0.83 ± 0.16 arbitrary units, *n* = 7, two-tailed *t*-test, *t* = 4.248, *P* = 0.00113). Using a functional rat cortical brain slice model, we find that postsynaptic muscarinic acetylcholine receptor function is dysregulated by aberrant amyloid beta-mediated activation of metabotropic glutamate receptor 5. Crucially, blocking metabotropic glutamate receptor 5 restores muscarinic acetylcholine receptor function and object recognition memory in 5XFAD transgenic mice. This indicates that the amyloid beta-mediated activation of metabotropic glutamate receptor 5 negatively regulates muscarinic acetylcholine receptor and illustrates the importance of muscarinic acetylcholine receptors as a potential disease-modifying target in the moderate pathological stages of Alzheimer’s disease.

## Teaser

Levels of muscarinic acetylcholine receptors were reduced in temporal cortex tissue of patients with Alzheimer’s disease with moderate pathology. Functional experiments in a complementary rat disease model showed impairment of muscarinic acetylcholine receptors was due to aberrant activation of the metabotropic glutamate receptor subtype 5. Results emphasize the importance of muscarinic acetylcholine receptors as a potential disease-modifying target.

## Graphical Abstract

**Graphical Abstract:**
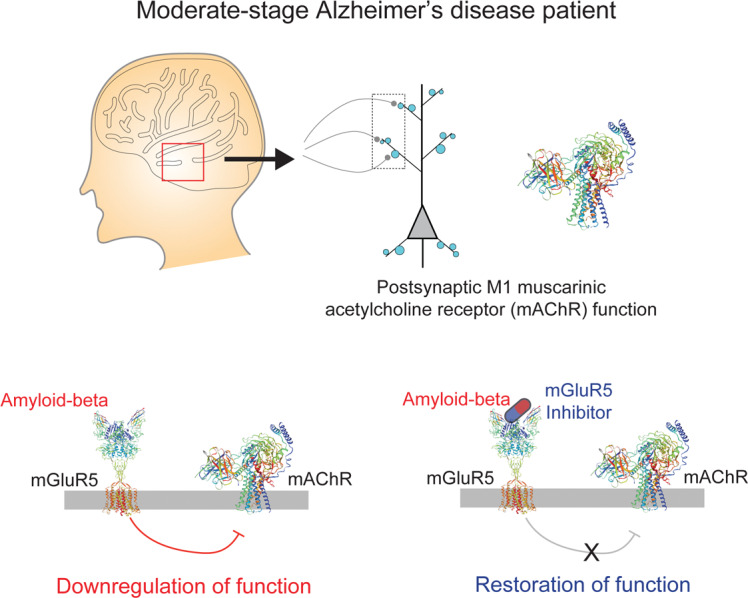

## Introduction

Alzheimer’s disease causes neurodegeneration in the brain and represents the major, and as yet incurable, form of dementia. A striking feature of the disease is the loss of cholinergic innervation of the cortex, which occurs alongside the pathological progression of Alzheimer’s disease. The ‘cholinergic hypothesis of Alzheimer’s disease’ has accordingly directed the development of clinically approved acetylcholinesterase inhibitors that function to enhance available acetylcholine in synapses. However, acetylcholinesterase inhibitors are fundamentally limited in their clinical efficacy (Bond *et al.*, 2012), indicating that the loss of acetylcholine alone does not account for the cholinergic dysfunction. Thus, whilst acetylcholine abundance is doubtlessly important, functional impairment of the acetylcholine receptor itself in the synapse may also play a role in Alzheimer’s disease. Therefore, it is of interest to determine whether muscarinic acetylcholine receptor (mAChR) dysfunction features in Alzheimer’s disease, and if so whether this receptor can be targeted as a disease-modifying strategy.

The M1 mAChR is a sub-family of G-protein-coupled mAChRs. They operate through a Gi/Gq signalling cascade, mobilizing Ca^2+^ from internal Ca^2+^ stores (Caulfield, 1993). The activation of mAChRs induces long-term synaptic plasticity in the perirhinal cortex, an area pivotal for novel object recognition memory (Warburton *et al.*, 2003). Indeed, blocking mAChRs causes deficits in object recognition memory (Warburton *et al.*, 2003). Given that this particular form of memory is highly sensitive to disruption in the early stages of Alzheimer’s disease (Dickerson *et al.*, 2017), and the perirhinal cortex is subject to volume loss in Alzheimer’s disease (Juottonen *et al.*, 1998), this provides an intriguing putative link between impaired mAChR function and memory deficits in Alzheimer’s disease.

At the molecular level, activation of mAChRs negatively regulates synaptic *N*-methyl-d-aspartate receptor (NMDAR) function (Jo *et al.*, 2010) and glycogen synthase kinase 3 signalling (De Sarno *et al.*, 2006), a key component in a mechanism that drives tauopathy in the brain (Hernandez *et al.*, 2012). Critically, M1 mAChR function is inhibited by aberrant activation of metabotropic glutamate receptor 5 (mGluR5) in the perirhinal cortex (Jo *et al.*, 2006). This is important as mGluR5 has been shown to act as a co-receptor for amyloid beta (Aβ) at the postsynaptic terminal (Um *et al*., 2013; Haas and Strittmatter, 2016), playing an intermediary role between Aβ and intracellular signalling cascades that lead to synapse loss and neurodegeneration (Um *et al*., 2013; Haas and Strittmatter, 2016). Therefore, mGluR5 may be involved in the pathological impairment of mAChR in the perirhinal cortex in Alzheimer’s disease.

To test this hypothesis, we first investigated whether M1 mAChR, the dominant form of mAChR in the brain, is aberrantly regulated in the moderate pathological stages of Alzheimer’s disease. We find a reduction in M1 mAChR protein expression in post-mortem temporal cortical tissue from patients with moderate-stage Alzheimer’s disease. We also find that Aβ oligomers facilitate the aberrant activation of mGluR5, consequently leading to weakening of postsynaptic M1 mAChR function in the perirhinal cortex. Furthermore, systemic infusion of an mGluR5-negative allosteric modulator [LSN2300979 (LSN)] rescues the otherwise impaired object recognition memory in the 5XFAD (familial Alzheimer’s disease) transgenic (TG) mouse model of Alzheimer’s disease pathology. Thus, our results suggest that inhibition of mGluR5 is beneficial for mAChR function and disease modification in moderate stages of Alzheimer’s disease.

## Materials and methods

### Post-mortem brain tissue

Human post-mortem brain tissue was provided by the South West Dementia Brain Bank (University of Bristol, UK), with local Research Ethics Committee approval. A total of 200 mg of non-specified temporal cortex samples from 14 patient brain tissue donations were used for this study (8 female and 6 male patients). The samples were categorized as ‘control’ or ‘moderate Alzheimer’s disease’. All control samples had a CERAD diagnosis (Mirra *et al.*, 1991) of ‘No Alzheimer’s disease’, and Braak staging (Braak and Braak, 1995) pathology of I–II. All moderate Alzheimer’s disease samples had a CERAD diagnosis of ‘possible/probable Alzheimer’s disease’, and Braak staging pathology of IV. No differences in age (control: 85 ± 2.63 years old, moderate Alzheimer’s disease: 84 ± 2.32 years old, *P*-value = 0.721, ‘data not shown’) or ‘post-mortem’ delay (control: 45.78 ± 4.78 h old, moderate Alzheimer’s disease: 44.28 ± 11.34 h, *P*-value = 0.209, ‘data not shown’) were detected between control and moderate Alzheimer’s disease groups. Human ‘post-mortem’ frozen samples were lysed at room temperature in sucrose/sodium dodecyl sulfate (SDS) buffer (lysis buffer) containing 1% SDS, 0.1 g/ml sucrose, 1 mM Ethylenediaminetetraacetic acid (EDTA), protease inhibitor cocktail at 1:10 (05892791001, Roche), phosphatase inhibitor cocktail at 1:100 (P5726, Sigma) and the serine/cysteine protease inhibitor phenylmethanesulfonyl fluoride at 1:100 (PMSF, P7626; Sigma). Approximately 100 mg of tissue were lysed in 500 μl of sucrose/SDS buffer. Samples were then centrifuged at 16 g for 20 min at 16°C. Pellets were discarded, and supernatants were collected in a fresh tube, constituting the stock solutions. Stock solutions were diluted 1:10 in lysis buffer to generate the working concentration sample. Samples were aliquoted and stored at −80°C until required. Protein levels were quantified using bicinchoninic acid (BCA) assay (Thermo Fisher Scientific). Following electrophoresis using sodium dodecyl sulphate-polyacrylamide gels, immunoblot analysis were performed using the relevant antibodies and visualized with chemiluminescence. The chemiluminescent signal was detected using the G:Box Chemi XT4 imaging system (Syngene, UK), and images were taken with GeneSys software (Syngene, UK). The following primary antibodies and dilutions were used: Anti-NR1 (1:1000, Upstate, 06-31; Matsuno *et al.*, 2015), Anti-GluA2 (1:1000, Millipore, MAB397; Park *et al.*, 2007; Peng *et al.*, 2015), Anti-mGluR1 (1:1000, ProteinTech, 19955-1-AP; Koochekpour *et al.*, 2012), Anti-mGluR5 (1:2000, Millipore, AB5675; Deschwanden *et al.*, 2011), Anti-M1 mAChR (1:200, Millipore, AB5164; Takamori *et al.*, 2007; Molina *et al.*, 2014) and Anti-postsynaptic density 95 (1:1000, Cell Signal, 3409; Martineau *et al.*, 2018; Subkhangulova *et al.*, 2018). The following secondary antibodies were used: Anti-mouse horseradish peroxidase (HRP) conjugate (1:4000, Upstate, 12-349) and Anti-rabbit HRP conjugate (1:4000, Millipore, 12-348). Data presented in the figures are cropped; uncropped western blot data are shown in Supplementary Fig. 1.

### Animals

All procedures involving animals used for *in vitro* experiments were performed in accordance with the UK Animals (Scientific Procedures) Act, 1986. All experimental protocols were approved by the University of Bristol Animal Welfare and Ethical Review Body. Animals were housed in groups and exposed to the 12-h light/12-h dark cycle. Animals were housed in controlled environmental conditions with food and water available *ad libitum*. Calcium imaging experiments were conducted using organotypic slices prepared from P7–8 male Wistar rats. Electrophysiology experiments were conducted using acute slices prepared from P25–35 male Wistar rats. Behavioural experiments were approved by the Institutional Animal Care and Use Committee of Kyung Hee University (Seoul, Republic of Korea). Mice were housed four per cage and were allowed access to water and food *ad libitum*. Animals were kept at a constant environment; temperature (23 ± 1°C) and relative humidity (60 ± 10%) under a 12-h light/dark cycle (lights on from 7:30 A.M. to 7:30 P.M.). Behavioural experiments were conducted using 6-month-old 5XFAD TG mice. These animals overexpress mutant amyloid precursor protein [K670N/M671L (Swedish), I716V (Florida) and V717I (London mutation)], along with mutant PS1 with M146L and L286V mutations. This results in the enhanced production of Aβ with a rapid onset, with Aβ typically expressed intraneuronally at 1–2 months of age (Oakley *et al.*, 2006).

### Electrophysiology

Whole cell recording for holding current was performed using acute perirhinal cortex slices (400 μm), which were perfused with artificial CSF (continuously bubbled with 95% O_2_/5% CO_2_) containing 124 mM NaCl, 3 mM KCl, 26 mM NaHCO_3_, 1.25 mM NaH_2_PO_4_, 2 mM CaCl_2_, 1 mM MgSO_4_ and 10 mM d-glucose. Recording electrodes (5–6 MΩ) containing potassium gluconate filling solution were used to patch and voltage clamp rhinal sulcus, layer II/III neurons. These were visualized using oblique contrast microscopy, through an Olympus BX51-WU microscope (Olympus, UK). The holding current recording experiments were perfused with MK-801 (10 μM) and tetrodotoxin (500 nM) in artificial CSF. The baseline of holding current was recorded for 10 min and then carbachol (CCh; 50 μM) applied for 5 min in the perfusate.

### Aβ preparation

The Aβ 1–42 peptide (Stratech, Ely, UK) was initially dissolved at a concentration of 1 mg/ml in 100% 1,1,1,3,3,3-hexafluoro-2-propanol (Sigma-Aldrich). This solution was then incubated for 1 h at room temperature, with occasional vortexing at a moderate speed. The solution was then sonicated for 10 min in a water bath sonicator. The 1,1,1,3,3,3-hexafluoro-2-propanol/peptide solution was next dried under a gentle stream of nitrogen gas. Then 100% dimethyl sulfoxide (DMSO) was then used to resuspend the peptide, which was then incubated at room temperature for 12 min with occasional vortexing. The final solution was aliquoted into smaller volumes and stored at −80°C. For a working solution, 500–1000 µl (depending on the final concentration to be used) Dulbecco's phosphate-buffered saline (D-PBS; Invitrogen, UK) was added to the peptide stock solution and incubated for 2 h at room temperature to allow for peptide aggregation. We have previously characterized this preparation to contain 1–5 nM of low-n oligomers (Whitcomb *et al.*, 2015).

### Organotypic brain slice culture

Perirhinal cortex organotypic slice cultures were prepared from 7- to 8-day-old male Wistar rats. After decapitation, the brain was immediately placed in ice-cold cutting solution (in mM: 238 sucrose, 2.5 KCl, 26 NaHCO_3_, 1 NaH_2_PO_4_, 5 MgCl_2_, 11 d-glucose and 1 CaCl_2_). A midsagittal section of the brain was made, the rostral and caudal parts were removed by single scalpel cuts 45° to the dorsoventral axis and each half was glued by its caudal end to a vibroslice stage (Campden Instruments, Sileby, UK). Slices (350 μm) that included perirhinal, entorhinal and temporal cortices were trimmed to create sections centred on the rhinal sulcus, containing layers I–V. Following washing, slices were plated on the top of semipermeable membrane inserts (Millipore Corporation, Bedford, MA, USA) within a six-well plate, which contained culture medium, comprised 78.8% minimum essential medium, 20% heat-inactivated horse serum, 25 mM HEPES, 10 mM d-glucose, 26 mM NaHCO_3_, 2 mM CaCl_2_, 2 mM MgSO_4_, 70 µM ascorbic acid, 1 µg/ml insulin, pH adjusted to 7.3 and 320–330 mOsm. The slices were then cultured in an incubator (35°C, 5% CO_2_) for 7–10 days *in vitro*. The medium was changed every 2 days. Neurons were transfected with GCaMP6 using a biolistic gene gun (Helios Gene-gun system; Bio Rad, USA) at days *in vitro* 3–4. Imaging assays were performed 3–4 days after transfection.

### Calcium imaging

Cultured perirhinal cortex slices were biolistically transfected with GCaMP6, the genetically encoded calcium indicator. Fluorescent images were captured using a BX51-WU microscope fitted with an ORCA-ER digital camera (Hamamatsu), at 1 frame/5 s. Following a 2 min baseline, 10 ml, 50 μM CCh was perfused onto the slice using a gravity-fed dropper system. Fluorescence intensity was quantified as a percentage of the normalized baseline, using Image J (National Institutes of Health, Bethesda, MD, USA).

### Object recognition

The experimental apparatus consisted of a black rectangular open field (25 cm × 25 cm × 25 cm). The novel object recognition task was carried out as described elsewhere (Li *et al.*, 2004; Barker and Warburton, 2011) with slight modifications. For the novel object location recognition test, mice were habituated to the open field in the absence of objects but with an internal cue on one of the four walls for 4 days. In the training phase, mice were replaced in the same box but now with two distinct objects. The objects consisted of a glass box and plastic cylinder. Mice were allowed to freely explore the environment and the objects for 5 min. After 3 h, mice were placed back in the rectangular environment for the testing phase. The two objects were again present, but one of the two objects were now displaced to a novel spatial location. Mice were allowed to freely explore the environment and the objects for 5 min. Time spent exploring the displaced and non-displaced objects were measured. Each group’s ability to recognize the novel location was determined by dividing the mean time exploring the novel object location by the mean of the total time exploring both objects during the test session. This value was multiplied by 100 to obtain a percentage preference for the novel object (*T*_novel_/*T*_total_ × 100, *T*_familiar_/*T*_total_ × 100). 5XFAD TG mice were treated with oral administration of LSN (30 mg/kg) or vehicle (Veh) (0.9% saline) for 8 days. On Days 4 and 5, animals from both groups were habituated to test environment. Following training on Day 6, animals were tested on Day 7, or in the 30-day interval trial training took place on Day 37 and testing took place on Day 38.

### Statistical analysis

No statistical methods were used to predetermine sample sizes. However, sample sizes were chosen based on previous studies published in the field by this laboratory and others. No data points were excluded from the analysis. Data pooled across slices are expressed as the mean ± SEM. Statistics were based on two-tailed Student’s *t* tests or one-way ANOVA followed by Tukey’s *post hoc* analyses for multiple comparisons, unless otherwise indicated. *P* < 0.05 was considered statistically significant.

### Data availability

The data pertaining to the findings described here are available from the corresponding author upon reasonable request.

## Results

### Loss of M1 mAChR protein in moderate Alzheimer’s disease temporal cortex

**Figure 1:**
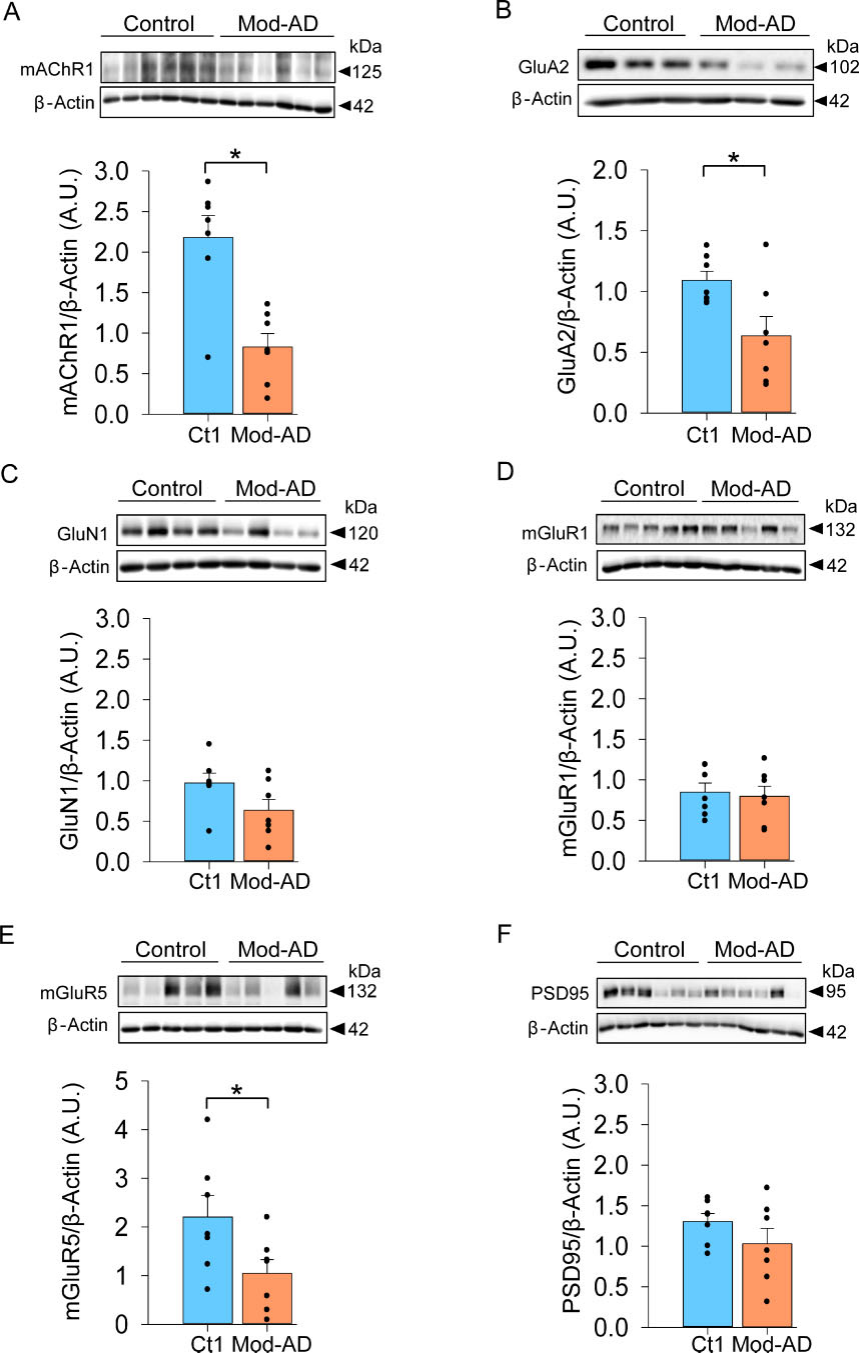
**Loss of M1 mAChR protein in human post-mortem mod-Alzheimer’s disease temporal cortex.** (**A**) mAChR1 protein levels are significantly reduced in the Mod-AD group (*N* = 7) compared to the control group (*N* = 7). (**B**) GluA2 protein levels are also significantly reduced in the Mod-AD group (*N* = 7) compared to the control group (*N* = 7). (**C**) GluN1 protein levels are not significantly different between the Mod-AD group (*N* = 7) compared to the control group (*N* = 7). (**D**) mGluR1 protein levels are not significantly different between the Mod-AD group (*N* = 7) and the control group (*N* = 7). (**E**) mGluR5 protein levels are significantly reduced in the Mod-AD group (*N* = 7) compared with the control group (*N* = 7). (**F**) Postsynaptic density-95 protein levels are not significantly different between the Mod-AD group (*N* = 7) and the control group (*N* = 7). Dots represent the value for each subject, and error bars represent the SEM. Significance level (*P < 0.05) was determined using an unpaired two-tailed Student’s *t*-test. Insets show cropped representative immunoblots, full length blots available in Supplementary Fig. 1. A.U. = arbitrary units.

Using temporal cortex brain tissue from moderate-stage (Mod-Alzheimer’s disease) and non-disease patient brain tissues (control), we determined total protein levels of key excitatory synaptic receptors. We found a significant reduction in M1 mAChR in the Mod-Alzheimer’s disease group [control: 2.17 ± 0.27 arbitrary units (A.U.), Mod-AD: 0.83 ± 0.16 A.U., two-tailed *t*-test, *t* = 4.248, *P* = 0.00113, Fig. 1A], indicating a loss of this receptor in moderate disease pathology. A similar effect was found in the GluA2 subunit of α-amino-3-hydroxy-5-methyl-4-isoxazolepropionic acid receptors (control: 1.09 ± 0.07 A.U., Mod-AD: 0.63 ± 0.16 A.U., two-tailed *t*-test, *t* = 2.597, *P* = 0.0234, Fig. 1B). Interestingly, no such loss was observed in GluN1 NMDARs (control: 0.97 ± 0.12 A.U., Mod-AD: 0.64 ± 0.13 A.U., two-tailed *t*-test, *t* = 1.875, *P* = 0.853, Fig. 1C), which is consistent with the widely accepted role NMDARs play in the pathophysiology of Alzheimer’s disease (Wang and Reddy, 2017). To determine whether the loss of mAChRs represented a generalized loss of G-protein-coupled receptors, we also assayed total levels of the related mGluR1 and mGluR5 subunits. Intriguingly, we found no difference in the levels of mGluR1 between the groups (mGluR1; control: 0.84 ± 0.11 A.U., Mod-AD: 0.76 ± 0.12 A.U., two-tailed *t*-test, *t* = 0.311, *P* = 0.761, Fig. 1D), but a significant reduction in mGluR5 (control: 2.2 ± 0.44 A.U., Mod-AD: 1.04 ± 0.28 A.U., two-tailed *t*-test, *t* = 2.196, *P* = 0.0485, Fig. 1E). Furthermore, we found no loss in levels of the postsynaptic density-95 protein, the prototypical postsynaptic protein and marker of postsynaptic integrity (control: 1.3 ± 0.09 A.U., Mod-AD: 1.03 ± 0.18 A.U., two-tailed *t*-test, *t* = 1.286, *P* = 0.223, Fig. 1F). Together, these data indicate a selective loss of mAChR, GluA2 and mGluR5 in the moderate pathological progression of Alzheimer’s disease.

### Aβ impairs mAChR function

**Figure 2:**
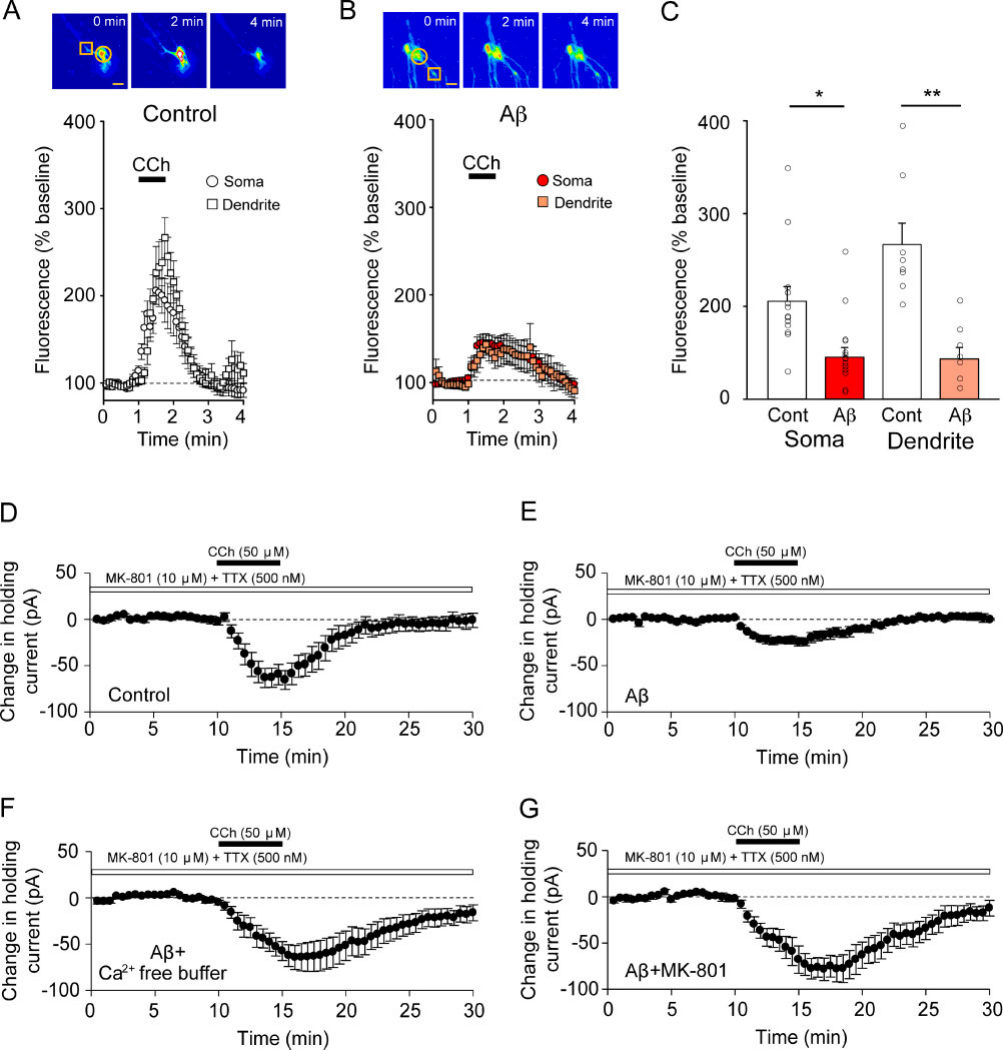
**Aβ impairs mAChR function in the perirhinal cortex.** (**A**) Quantification of GCaMP6-mediated Ca^2+^ fluorescence in transfected neurons from perirhinal cortex organotypic culture. Following a 1-min baseline, CCh is perfused onto slices. Whilst this causes a robust increase in fluorescence in control slices, (**B**) a much smaller magnitude of fluorescence increase is found in slices treated with Aβ. (**C**) Quantification in peak fluorescence change induces by CCh perfusion. Aβ-Treated slices had statistically smaller peak fluorescence values in both soma and dendrites compared with control slices. Bars represent ± SEM. (**D**) Change in holding current induced by CCh perfusion, normalized to a 10-min baseline recording. Whilst CCh induces robust change in holding current, (**E**) CCh induces a modest change in holding current in slices pre-exposed to Aβ prior to recording (**F**). This impairment of holding current change by Aβ is ameliorated in slices pre-exposed to Aβ in Ca^2+^-free artificial CSF (**G**) and in slices pre-exposed to Aβ in the presence of the NMDAR blocker MK-801. Figures represent pooled data across separate experiments, with error bars representing ± SEM. Significance level (*P < 0.05) was determined by using an unpaired Student’s *t*-test. Scale bar indicates 10 μm.

Organotypic cultured perirhinal cortex slices were biolistically transfected with the genetically encoded Ca^2+^ indicator GCaMP6 (Chen *et al.*, 2013), and we measured the magnitude of fluorescence induced by Ca^2+^ mobilization on ligand activation of mAChRs. Bath application of CCh (50 μM), the mAChR agonist, induced a robust and transient increase in Ca^2+^ both in the soma (open symbols, peak fluorescence intensity as a % of baseline, 205 ± 16%, *n* = 13 cells from 10 slices) and dendrites (closed symbols, peak fluorescence intensity as a % of baseline 266 ± 23%, *n* = 8 dendrites from eight slices; Fig. 2A). However, in slices treated for 2 h with 1–5 nM Aβ oligomers, the CCh-induced Ca^2+^ signal was significantly reduced (open symbols, soma peak fluorescence intensity as a % of baseline 145 ± 10%, *n* = 16 cells from 12 slices; closed symbols, dendrite peak fluorescence intensity as a % of baseline 143 ± 12%, *n* = 8 dendrites from 9 slices; Fig. 2B). The magnitude of CCh-induced fluorescence was reduced in Aβ-treated slices compared with control slices, in both the soma (control versus Aβ, two-tailed *t*-test, *t* = 3.320, *P* = 0.00258) and dendrites (control versus Aβ, two-tailed *t*-test, *t* = 4.847, *P* = 0.000213; Fig. 2C). This suggests that Aβ oligomers rapidly impair mAChR function.

### Weakening of postsynaptic mAChR function by Aβ-induced pathophysiology

To confirm this, we next analysed postsynaptic mAChR-mediated holding current. Neurons were voltage clamped at −70 mV, and the injected current required to maintain the holding current was monitored, before and after application of CCh (50 μM). Perfusion of CCh caused a rapid increase in negative holding current, indicative of activation of mAChRs and consequent inward current due to Ca^2+^ release from intracellular stores (peak depression −62 ± 14 pA, *n* = 5; Fig. 2D). In slices treated for 2 h with 1–5 nM Aβ oligomers, there was a significant reduction in holding current change in response to CCh (peak depression −24 ± 3 pA, *n* = 5; Fig. 2E; control versus Aβ *P* = 0.0368), indicating that postsynaptic mAChRs were no longer activated to the same extent as that observed in the control condition. This suggests that Aβ dysregulates postsynaptic mAChR function, possibly through the impairment of classical G-protein-coupled receptor-mediated signal cascades or through the modulation of the expression of the receptor itself. Since the aberrant mobilization of Ca^2+^ by Aβ is known to trigger neurotoxicity in cell models (Demuro *et al.*, 2005), we wondered whether extracellular Ca^2+^ was important in this effect. To test this, slices were exposed for 2 h with 1–5 nM Aβ oligomers in Ca^2+^-free artificial CSF and CCh-induced holding current change was examined. Interestingly, we found that under these conditions, Aβ had no impairing effects (−63.8 ± 14.3 pA, *n* = 10; Fig. 2F). This suggests that Aβ-mediated Ca^2+^ mobilization causes mAChR dysfunction. The NMDAR is a central postsynaptic Ca^2+^ regulator and a key conduit for Aβ-induced neuronal dysfunction (Wang and Reddy, 2017). Furthermore, the inhibition of NMDAR is beneficial in Alzheimer’s disease and object recognition memory in animal models of the disease (Romberg *et al.*, 2012). Therefore, we hypothesized that Aβ-induced impairment of mAChR function occurs in an NMDAR-dependent manner. When slices were co-exposed to 1–5 nM Aβ oligomers and MK-801 (10 μM), the NMDAR channel blocker, in normal Ca^2+^-containing artificial CSF for 2 h, we found that Aβ did not impair the inward-holding current change (−77.2 ± 12.7 pA, *n* = 7; Fig. 2G). Together, these data suggest that both extracellular Ca^2+^ and activation of NMDARs are essential for Aβ-mediated mAChR dysfunction.

### Aβ inhibits postsynaptic mAChR function by aberrant activation of mGluR5

**Figure 3:**
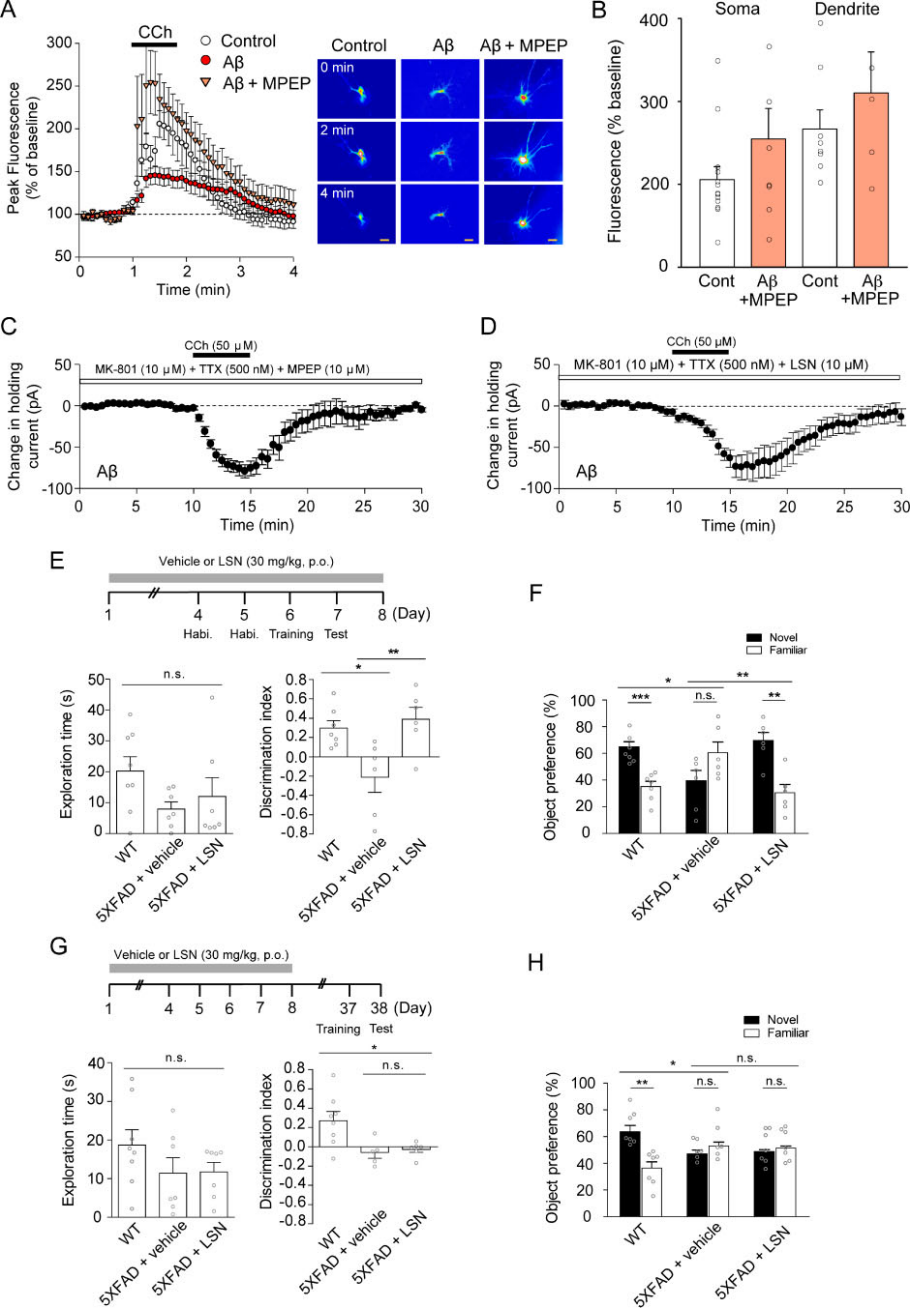
**Inhibition of mGluR5 prevents the Aβ-mediated impairment of mAChR function and restores cognitive function.** (**A**) Quantification of GCaMP6-mediated Ca^2+^ fluorescence in transfected neurons from perirhinal cortex organotypic culture. Whilst there was only a modest increase in fluorescence in Aβ-treated slices, there was a robust increase in fluorescence in Aβ + MPEP-treated slices. Scale bar indicates 10 μm. (**B**) There was no statistically significant difference in peak fluorescence intensity induced by CCh perfusion between control and Aβ + MPEP-treated slices. (**C**) Change in holding current induced by CCh perfusion. A robust change in holding current was induced by CCh in slices treated with Aβ + MPEP (**D**) and in slices treated with Aβ + LSN. Holding current data illustrated in **C** and **D** were recorded from acute perirhinal cortex slices from p25–35 Wistar rats. (**E**) Quantification of behavioural assessment of object recognition memory in 5XFAD mice, comparing WT, vehicle (5XFAD + vehicle) and LSN-treated (5XFAD + LSN) animals. (**F**) Quantification of object preference between novel and familiar objects, comparing the animal treatment groups. Whilst WT animals showed a clear preference for the novel object, 5XFAD + vehicle animals did not. 5XFAD + LSN animals showed a similar novel object preference to WT animals. (**G**) Quantification of behavioural assessment of object recognition memory across the experimental conditions outlined above, with a delay of 30 days introduced between vehicle or LSN treatment and the training and testing. (**H**) Whilst WT animals displayed a preference for novel objects, neither the vehicle-treated nor LSN-treated animals did. Significance level (*P < 0.05; **P < 0.01; ***P < 0.001) was determined by using a one-way ANOVA with *post hoc* Tukey analysis. Dots in **E** and **G** represent the value for each subject.

The function and expression of mAChRs is regulated by a number of different cellular processes, including phosphorylation and trafficking (van Koppen and Kaiser, 2003). A previous study also found that juvenile rat mAChR function was down-regulated by aberrant activation of mGluR5, a group I metabotropic glutamate receptor (Jo *et al.*, 2006). Furthermore, evidence suggests that mGluR5 and the synergistically regulated NMDAR function may be involved in Aβ-mediated pathology and deficits to object recognition memory in Alzheimer’s disease (Alagarsamy *et al.*, 2001; Renner *et al.*, 2010; Hu *et al.*, 2014; Hamilton *et al.*, 2016). We therefore hypothesized that the weakening of mAChR function in Alzheimer’s disease pathology is underpinned by the aberrant interplay between NMDAR and mGluR5 activation. We applied the mGluR5 antagonist 2-methyl-6-(phenylethynyl)pyridine (MPEP, 50 μM) to slices and then exposed those slices for 2 h to 1–5 nM Aβ oligomers. We found that, in contrast to Aβ treatment alone (Fig. 2B), inhibition of mGluR5 restored CCh-induced Ca^2+^ signalling (triangle symbols, soma peak fluorescence intensity as a % of baseline 254 ± 37%, *n* = 7 cells from five slices/four animals; dendrite peak fluorescence intensity as a % of baseline 310 ± 49%, *n* = 5 dendrites from 5 cells; Fig. 3A). The magnitudes of somatic and dendritic Ca^2+^ on CCh application did not differ between the Aβ with MPEP group and untreated controls (soma: control versus Aβ + MPEP, two-tailed *t*-test, *t* = −1.432, *P* = 0.169; dendrite: control versus Aβ + MPEP, two-tailed *t*-test, *t* = −0.903, *P* = 0.386; Fig. 3B). Consistent with these findings, we found that the application of MPEP or the mGluR5-negative allosteric modulator LSN (10 μM) prevented the Aβ-mediated impairment of CCh-induced inward-holding current change (peak depression −79 ± 7 pA, *n* = 6, control versus Aβ + MPEP *P* = 0.3; Fig. 3C; peak depression −71.5 ± 14.5 pA, *n* = 6; Aβ + LSN; Fig. 3D). Together, these data indicate that the impairment to mAChR function that is induced by Aβ occurs in unison with the aberrant activation of mGluR5, and this is causally linked.

### An mGluR5-negative allosteric modulator restores novel object recognition memory in 5XFAD TG mouse

Since the inhibition of mGluR5 was critical to maintain mAChR function, and the function of mAChRs is important in object recognition memory (Warburton *et al.*, 2003), we wondered what role mGluR5 might play in the widely reported dysregulation of cognitive function by Aβ (McGowan *et al.*, 2006). We therefore examined novel object recognition memory in the 5XFAD TG mouse model. Whilst there were no differences in exploration time between experimental groups [one-way ANOVA (Tukey’s *post hoc* test) *P* = 0.1789, wild type (WT): 20.23 ± 4.665 s, *n* = 8; TG-Veh: 7.946 ± 2.279 s, *n* = 7; TG-LSN: 12.01 ± 6.068 s, *n* = 7, Fig. 3E], there was a significant impairment in object discrimination performance in the 5XFAD mice compared with WT mice [0.2971 ± 0.07748 (*n* = 7) versus TG-Veh: −0.2122 ± 0.1565 (*n* = 6), *P* < 0.05, Fig. 3E]. Critically, this deficit was ameliorated in 5XFAD mice treated with LSN [TG-Veh versus TG-LSN: 0.3889 ± 0.1234 (*n* = 6), *P* < 0.001, Fig. 3E]. Indeed, whilst the WT animals showed an exploration preference for the novel object (novel: 64.860 ± 3.874% versus familiar: 35.140 ± 3.874% object preference, Fig. 3F), the 5XFAD mice displayed no such preference (novel: 39.390 ± 7.827% versus familiar: 60.610 ± 7.827% object preference, Fig 3F). Importantly, the 5XFAD animals treated with LSN displayed similar object preferences to the WT control animals (novel: 69.450 ± 6.169% object preference versus familiar: 30.550 ± 6.169% object preference, Fig. 3F).

We wondered whether the restorative effects of mGluR5 antagonism reflected a fundamental amelioration of the disease state. To test this, we administered the same drug regime but introduced a 30-day interval period between the last drug infusion and training and testing. Whilst there were no differences in exploration time between experimental groups [one-way ANOVA (Tukey’s *post hoc* test) *P* = 0.275, WT: 18.69 ± 3.975 s, *n* = 8; TG-Veh: 11.39 ± 3.994 s, *n* = 7; TG-LSN: 11.70 ± 2.472 s, *n* = 7, Fig. 3G], there was a significant impairment in object discrimination performance across the groups [one-way ANOVA (Tukey’s *post hoc* test) *P* = 0.0102, WT: 0.2716 ± 0.09399, *n* = 8; TG-Veh: −0.06025 ± 0.05867, *n* = 5; TG-LSN:-0.02718 ± 0.03122, *n* = 6]. However, there was no difference in discrimination performance between the 5XFAD Veh mice and those treated with LSN (*P* = 6.134). Whilst the WT animals showed an exploration preference for the novel object (novel: 63.580 ± 4.699% object preference versus familiar: 36.420 ± 4.699% object preference, Fig. 3H), the 5XFAD mice displayed no such preference (novel: 46.990 ± 2.933% object preference versus familiar: 53.010 ± 2.933% object preference, Fig 3H). Importantly, the 5XFAD animals treated with LSN also showed no preference (novel: 48.640 ± 1.561% object preference versus familiar: 51.360 ± 1.561% object preference, Fig. 3H). This indicates that whilst downregulation of mGluR5 function is a putative means by which cognitive function can be restored, the pathology is underpinned by the constitutive aberrant activation of the receptor and could therefore require sustained inhibition for restorative effect.

## Discussion

Our protein assay data from moderate Alzheimer’s disease post-mortem brain tissue indicates a reduction in M1 mAChR. This is consistent with a dysfunction of the cholinergic transmission system occurring in the mild cognitive impairment phase of the moderate pathological stages of Alzheimer’s disease (Haense *et al.*, 2012). On the surface, our findings run counter to previous reports describing no change (Mash *et al.*, 1985) and an increase (Overk *et al.*, 2010) in M1 mAChRs in Alzheimer’s disease human tissue. Importantly, these studies assayed tissue from patients with late-stage Alzheimer’s disease, reinforcing our conclusion that mAChR dysregulation is a feature of the progressive pathology of Alzheimer’s disease, and our present findings reflect moderate-stage effects. Our postnatal and juvenile rat model studies are indicative of the possible early events of Aβ-mediated dysfunction of mAChR. With this in mind, our results raise the question as to whether the reduction in M1 mAChR is a consequence of pathophysiology and underlies cognitive impairment. mAChR-mediated synaptic transmission plays a key role in the cellular mechanism of object recognition in the rat perirhinal cortex (Warburton *et al.*, 2003). Interestingly, we previously found that mGluR5 has a dominant role over M1 mAChR in this brain region in rats (Jo *et al.*, 2006). Since M1 mAChR and mGluR5 share the same Gi/Gq-coupled signalling mechanisms, the aberrant activation of mGluR5 function may dominantly sequester G proteins, resulting in a lack of available G-protein coupling with M1 mAChR. An alternative intriguing explanation is that Aβ disrupts M1 mAChR regulatory machinery, breaking down the stabilization and recycling processes of mAChR to the synapse (Gainetdinov *et al.*, 2004). Thus, it is plausible that Aβ may cause weakening of M1 mAChR function through mGluR5 activity and destabilization of mAChR. However, it is not known whether and/or how aberrant activation of mGluR5 function affects the expression of M1 mAChR in the progression of Alzheimer’s disease.

Considering the slow progress in translating basic characterization of Alzheimer’s disease pathophysiology to effective symptom and disease-modifying therapies, new targets are required. Synapses serve as sites where key pathological changes occur in Alzheimer’s disease (Selkoe, 2002). Defects in synaptic transmission and dendritic spine structure are well-associated with the progression of pathological events that greatly contribute to the destabilization of synaptic contacts and therefore are likely to have a significant impact on memory circuits (Cochran *et al*., 2014). More importantly, these changes occur at early stages of the disease, a period now considered to be critical for successful therapeutic interventions (Korolev, 2014). Within this context, our key finding is that Aβ drives the weakening of postsynaptic M1 mAChR function by aberrantly activating mGluR5. Since mAChR functions to affect a number of signal cascades, weakening of postsynaptic mAChR may have fundamental pathophysiological consequences, likely impacting on synaptic function and structure. For instance, weakening of mAChR may lead to aberrant activation of NMDAR in the perirhinal cortex (Jo *et al.*, 2010). Therefore, Aβ-mediated weakening of mAChR could also link to the regulation of a glycogen synthase kinase 3 signalling, one of the major Alzheimer’s disease-related synapse-weakening molecules (Jo *et al.*, 2011; Regan *et al*., 2015).

Within the limited therapeutic options available for Alzheimer’s disease, memantine is a widely used NMDAR blocker. We previously reported that memantine restored object recognition memory by blocking false memory in the TG CRND8 mouse model of Alzheimer’s disease (Romberg *et al.*, 2012). This showed that the aberrant activation of NMDARs causes pathophysiological synaptic plasticity and may dysregulate cellular mechanisms of object recognition memory in Alzheimer’s disease. Interestingly, our current study showed that Aβ-mediated weakening of mAChR function was Ca^2+^ mobilization and NMDAR dependent. This suggests the notion that Aβ inhibits mAChR function via either mGluR5 or through synergistic regulation between the mGluR5 and NMDARs. Since mGluR5 antagonism alone rescues mAChR function and object recognition memory, tight control of mGluR5 function will be an advantageous for the maintenance of mAChR function and preventing the progress of pathology and enhancing cognitive performance, as we have shown in this study.

Though previous studies have demonstrated that a broad conceptual role of mGluR5 antagonism is the restoration of memory function in TG mouse models of Alzheimer’s disease (e.g. Hamilton *et al.*, 2016), our study provides a framework for these effects: the aberrant activation of mGluR5 impairing postsynaptic mAChR function, where through blocking mGluR5, postsynaptic mAChR function and object recognition memory in Alzheimer’s disease models can be restored. Thus, we postulate that mGluR5-mediated dysfunction of postsynaptic mAChRs underlies a potential cellular mechanism of object recognition memory deficit in Alzheimer’s disease. This positions mAChR as a further potential therapeutic target when considering how to prevent acetylcholine-signalling decline in the developing pathology of Alzheimer’s disease.

## Supplementary material

Supplementary material is available at *Brain Communications* online.

## Supplementary Material

fcaa058_Supplementary_Data

## Abbreviations

Aβ =: amyloid beta
A.U. =: arbitrary units
CCh =: carbachol
FAD =: familial Alzheimer’s disease
LSN =: LSN2300979
mAChRs =: muscarinic acetylcholine receptors
mGluR5 =: metabotropic glutamate receptor 5
MPEP =: 2-methyl-6-(phenylethynyl)pyridine
NMDAR =: *N*-methyl-d-aspartate receptor
TG =: transgenic
Veh =: vehicle
WT =: wild type
