# Supplementary material for: M1 muscarinic acetylcholine receptor dysfunction in moderate Alzheimer’s disease pathology
Source: Brain Commun. 2020 May 12;2(2):fcaa058. doi: 10.1093/braincomms/fcaa058 (PMC7391992; doi:10.1093/braincomms/fcaa058)
Supplement: fcaa058_Supplementary_Data [file fcaa058_supplementary_data.pdf]

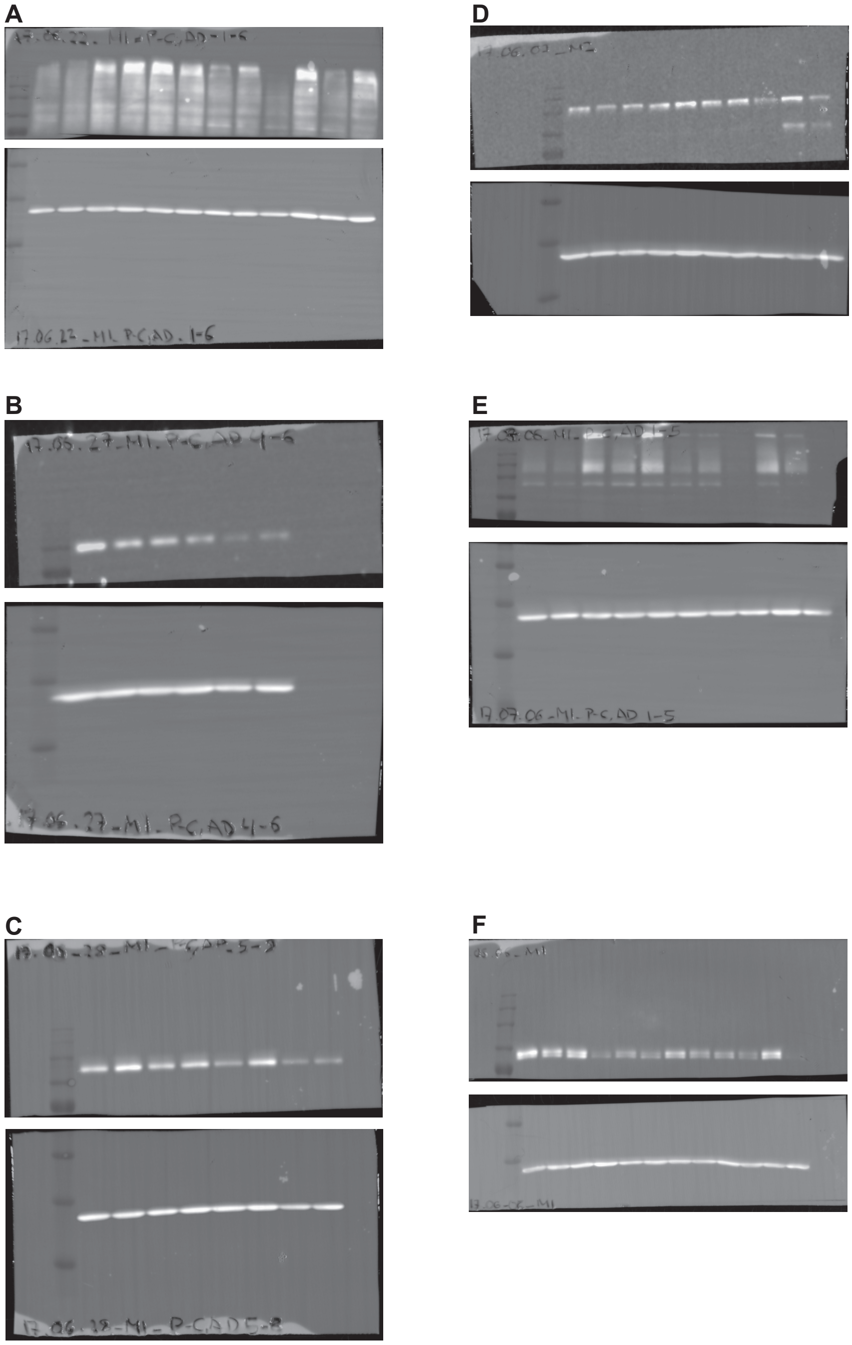

Supplementary Figure 1. The corresponding full lengths blots for the cropped blot data illustrated in Figure 1. The full-length blots have been cut to allow for the simultaneous acquisition of the protein of interest (top section) and the corresponding β-actin bands. Molecular weight was determined by the color prestained protein standard broad range 11-245 kDA (New England Biolabs P7712) visible in all blots. The full-length blots correspond to the same panels in Figure 1, such as the protein of interest is (A) mAChR1, (B) GluA2, (C) GluN1, (D) mGluR1, (E) mGluR5 and (F) PSD-95.
